# Abdominal lymphangioleiomyomatosis in a man presenting with gastrointestinal hemorrhage as the first manifestation: a case report

**DOI:** 10.3389/fmed.2024.1420414

**Published:** 2024-12-17

**Authors:** Ying Zi, Yuchen Shi, Rongjie Shi

**Affiliations:** ^1^The First Affiliated Hospital of Dali University, Dali, Yunnan, China; ^2^The First Affiliated Hospital of Kunming Medical University, Kunming, Yunnan, China; ^3^Department of Gastroenterology, The First Affiliated Hospital of Dali University, Dali, Yunnan, China

**Keywords:** lymphangioleiomyomatosis, gastrointestinal hemorrhage, abdomen, male, case report

## Abstract

Lymphangioleiomyomatosis (LAM) is a rare, low-grade malignant condition that typically affects women of childbearing age and primarily involves the lungs. While cases involving males and affecting the gastrointestinal tract are exceedingly uncommon. This report discusses an unusual case of abdominal LAM in a male patient with gastrointestinal hemorrhage. The patient, a 70-year-old man, had been experiencing recurrent abdominal pain, occasional black stools, dizziness, and fatigue for over a month before being admitted to the hospital. Diagnostic electronic gastroscopy identified ulcers in the gastric and duodenal bulb with hemorrhage. An abdominal CT scan revealed multiple cystic foci in the retroperitoneum and mesentery, but no masses were found. Despite receiving medical treatment, the patient continued to have black stools and eventually underwent laparoscopic distal subtotal gastrectomy. The pathological results of the excised distal gastric specimens showed LAM-like lesions in the submucosal layer of the pylorus, mesentery of the side of the lesser curvature of the stomach, and small intestine, leading to a diagnosis of abdominal LAM. However, even after the surgery, the patient still experienced recurrent black stools and developed new symptoms of chest tightness and shortness of breath. A follow-up chest CT revealed bilateral pleural effusion and multiple lung cysts, indicating a worsening condition. The patient was then prescribed oral Sirolimus, which resulted in an improvement in symptoms, including black stool, chest tightness, and shortness of breath. This case report provides a detailed account of the progression of an unusual gastrointestinal LAM case and suggests that a combination of surgery and Sirolimus may be effective in managing the condition.

## Introduction

Lymphangioleiomyomatosis (LAM) was first discovered and identified by Burrell in 1937 (1), and the first case reported in the Chinese mainland was in 1983 (2). LAM is a rare condition characterized by abnormal proliferation of smooth muscle cells around the lymphatic ducts, classified under the family of PEComas (3). The most common site of LAM is the lung, accounting for about 90% of all cases. Pulmonary LAM can cause progressive cystic changes in the lung, pneumothorax, chylothorax, and can lead to respiratory failure and ultimately death (4). LAM in the abdomen is generally rare, and gastrointestinal LAM is even rarer. In these cases, smooth muscle cell proliferation within the lymphatic system of the gastrointestinal tract forms tumor masses in the corresponding bowel wall areas (5). These lesions, typically multinodular in the gastrointestinal tract, often manifest as bleeding. Additionally, according to the published literature, gastrointestinal lymphangiomatosis mainly presents with non-specific chronic symptoms, such as abdominal pain, weakness, weight loss, abdominal distension, and, rarely, pitting edema due to hypoproteinemia, gastrointestinal bleeding, or obstruction (6). It is also noted that abdominal LAM can form large masses, but primary abdominal LAM typically shows non-specific imaging features apart from space-occupying lesions (7). This report highlights a rare instance of primary abdominal LAM in a male patient characterized by gastrointestinal bleeding to enhance the understanding of its clinical diagnosis and treatment.

## Case presentation

### Clinical history and the results of auxiliary examination in the first admission

A 70-year-old man was admitted to the hospital on 24 April 2023, with a month-long history of recurrent abdominal pain, intermittent black stools, dizziness, and fatigue. Upon admission, his physical examination showed a body temperature of 36.9°C, a heart rate of 80 beats per min, a respiratory rate of 20 breaths per min, and a blood pressure of 96/57 mm Hg. He exhibited noticeable pallor in his skin, mucous membranes, and eyelid conjunctiva, indicative of anemia. The patient appeared mentally weak. His lung examination revealed clear respiratory sounds bilaterally, and his heart rhythm was regular. His abdomen was soft and flat, with mild tenderness and active bowel sounds throughout, but there was no rebound pain, muscle tension, or shifting dullness. He had not experienced significant weight changes or body swelling since the onset of his symptoms. Prior to this illness, the patient has a 50-year smoking history and smoked about 20 cigarettes per day, besides this, he had been in good health with no history of diseases or medications and no known genetic disorders in his family history.

**FIGURE 1:**
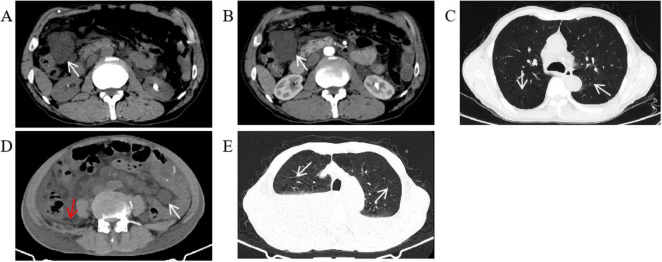
Abdominal and chest CT. **(A)** There were multiple round cystic hypodense shadows of different sizes in the retroperitoneum and mesentery. The white arrow points to the largest cystic hypodense shadows located in the right upper quadrant, about 4.2 cm × 3.4 cm in size and 10.8 Hu of density. **(B)** The largest cystic hypodense shadows saw no obvious enhancement on the contrast scan, 10.9 Hu of density. **(C)** The transparency of the two lung fields increased. The two lungs were scattered with cystic translucent shadows whose diameters were between 0.3 and 1.0 cm. **(D)** There were multiple round cystic hypodense shadows of different sizes in the retroperitoneum and mesentery. The white arrow points to the largest cystic hypodense shadows located in the left lower quadrant, about 2.5 cm × 2.1 cm in size and 10.3 Hu of density. A small amount of abdominopelvic effusion can be seen as the red arrow points. **(E)** The transparency of the two lung fields increased, and the two lungs were scattered with cystic translucent shadows as the arrow points. Bilateral pleural effusion can be seen with high-density liquid level.

**FIGURE 2:**
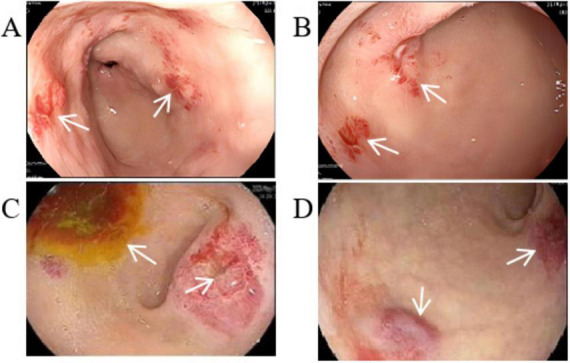
Gastroscopic image. **(A)** Multiple ulcerative lesions in the gastric antrum. **(B)** Mucosal erosion ulcer in the duodenal bulb. **(C)** Ulcerative lesions with enlarged surface and active bleeding. **(D)** Mucosal bulge in the duodenal bulb with surface congestion.

We performed several additional tests to evaluate the patient’s overall health. His blood routine indicated moderate anemia with a red blood cell (RBC) count of 3.42 × 10^12^/L while hemoglobin (HGB) was 65 g/L. The fecal occult blood test came back strongly positive, indicating possible hemorrhage. However, the digital rectal examination did not reveal any abnormalities, and tumor markers were negative. An abdominal CT scan showed multiple cystic hypodense shadows of varying sizes in the retroperitoneum and mesentery, with the largest measuring 4.2 cm × 3.4 cm and a density of 10.8 HU (Figure 1A), located in the right upper quadrant. This lesion did not show significant enhancement on the contrast scan, remaining at a density of 10.9 HU (Figure 1B). The chest CT revealed increased transparency in the lung fields, with scattered cystic translucent shadows in both lungs (Figure 1C). Electronic gastroscopy showed multiple ulcerative lesions in the gastric antrum, covered with white fur and surrounded by congested, bleeding mucosa, each measuring about 0.5 cm in diameter. The anterior and posterior walls of the duodenal bulb showed mucosal erosion, and a small, actively bleeding ulcer was found at the center of this erosion, with a congested bulge on the side of the stomach’s lesser curvature (Figures 2A, B). Biopsy samples from the gastric antrum and duodenal bulb revealed mild chronic inflammation in the gastric antrum mucosa with abundant inflammatory exudates. The duodenal bulb’s lamina propria showed congestion with infiltrations of lymphocytes, plasma cells, and medium neutrophils. Based on these histopathological findings, the patient was diagnosed with chronic gastritis (specifically, chronic superficial gastritis in the zero phase), as well as stomach and duodenal ulcers with bleeding (specifically, superficial ulcers in the active stage).

Based on his diagnosis, the patient received treatments aimed at managing his symptoms. These included suppressing gastric acid with Omeprazole (40 mg daily via intravenous drip), protecting the mucosa through oral administration of L-Glutamine and Sodium Gualenate Granules (0.67 g daily), stopping bleeding with Octreotide (0.6 mg daily, administered intravenously at 2.5 ml/h) and Carbazochrome Sodium Sulfonate Injection (80 mg daily via intravenous drip), and replenishing blood volume with red blood cell suspension through intravenous drip. Despite these interventions, the patient continued to experience black stools, indicating ongoing active bleeding in the digestive tract. A follow-up blood test on 15 May 2023, showed a RBC count of 4.34 × 10^12^/L and a hemoglobin level of 94 g/L. A second gastroscopy revealed that the ulcerative lesions in the gastric antrum had enlarged, with the mucosa showing an orange and red coloration and signs of active bleeding. Additionally, the area from the duodenal bulb to the descending part was covered with mucosal bulges, and the surface was infused with blood (Figures 2C, D).

After careful consideration of the patient’s ongoing active gastrointestinal bleeding and lack of response to conservative medical treatments, it was determined that surgery was necessary. Prior to the surgery, thorough evaluations were conducted to rule out any contraindications, including coagulation function tests, infectious disease screenings, and reviews of the previous surgical history and drug allergies. Consequently, a laparoscopic distal subtotal gastrectomy was performed on 16 May 2023. During the surgery, a bloody mass was observed in the gastric antrum, along with multiple small, unruptured bloody masses in the mesentery. The procedure involved clamping and cutting the duodenum 3 cm below the pylorus and carefully dissecting the associated arteries and veins. The lesser curvature of the stomach was mobilized upstream, and approximately one-third of the greater curvature was dissociated from left to right. The distal two-thirds of the stomach were then clamped and severed. To maintain digestive continuity, the jejunum was anastomosed laterally to the posterior wall of the stomach.

**FIGURE 3:**
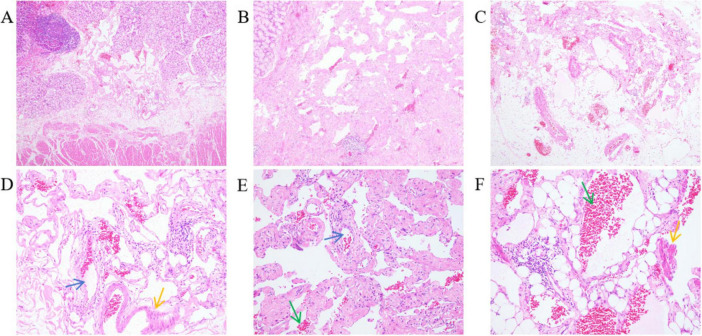
The mesentery of the duodenum and pylorus and the mesentery showed dilated lacuna-like structures (the blue arrow points) of varying sizes, covered with a single layer of flat epithelium. Pink lymphatic fluid was found in the lumen (the green arrow points). Proliferative smooth muscle cells (the yellow arrow points) around the focal lumen were observed. HE stains; **(A–C)** are 40× magnification; **(D–F)** are 200× magnification. **(A)** Submucosal layer of the pylorus. **(B)** Mesentery of the side of the lesser curvature of the stomach. **(C)** Mesentery of the small intestine. **(D)** Dilated lymphatic vessels and proliferative smooth muscle cells in the submucosal layer of the pylorus. **(E)** Dilated lymphatic vessels and lymphatic fluid in the mesentery of the side of the lesser curvature of the stomach. **(F)** Proliferative smooth muscle cells and lymphatic fluid in the mesentery of the small intestine.

**FIGURE 4:**
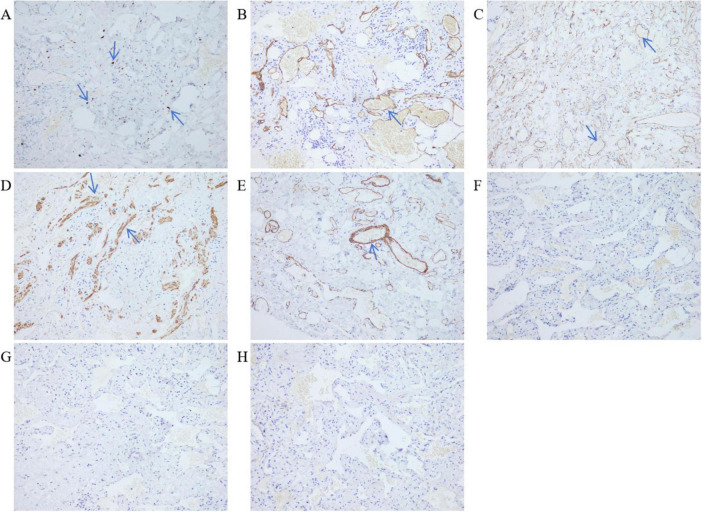
Immunohistochemistry of duodenal tissue. LDP staining; 200× magnification. **(A)** Ki67 (+5%). **(B)** Lymphatic vessel D2-40 (+). **(C)** Blood vessels CD34 (+). **(D)** Smooth muscle of the tube wall Desmin (+). **(E)** Smooth muscle of the tube wall SMA (+). **(F)** HMB-45 (–). **(G)** Melan-A (–). **(H)** S-100 (–).

The excised distal gastric and small intestinal mucosa were sent for examination. Visible to the naked eye were nodular lesions of various sizes in the excised tissue, with the largest having a diameter of 1.6 cm. Pathological biopsy analysis revealed LAM-like lesions in the submucosal layer of the pylorus, the mesentery adjacent to the lesser curvature of the stomach, and the small intestine (Figure 3). These lesions were determined to be T1NxM0 in grade and phase II in stage. Immunohistochemical results of duodenal tissue showed Ki67 (+5%), D2-40 (+), CD34 (+), Desmin (+), SMA (+), S-100 (−), Melan-A (−), HMB-45 (−) (Figure 4). Based on a thorough evaluation of the abdominal CT findings and the pathology biopsy results, the patient was definitively diagnosed with abdominal LAM. He showed signs of improvement post-surgery and was discharged from the hospital on 30 May 2023.

### Clinical history and the results of auxiliary examination in the second admission

On 3 October 2023, the patient was readmitted and reported experiencing chest tightness, and shortness of breath, besides the symptoms from his last hospitalization. The physical examination revealed persistent pallor in his skin, mucous membranes, and eyelid conjunctiva, indicating anemia. He still appeared mentally weak. Upon examination of the lungs, decreased respiratory sounds were noted, particularly in the right lung. His heart rhythm was regular, and scars from previous surgery were visible on his abdomen. He also reported mild tenderness in the epigastrium, especially below the xiphoid process. Active bowel sounds were present throughout the abdomen, and there were no signs of rebound pain or muscle tension, and shifting dullness was negative. He had gained 2 kg since his last discharge. Blood tests indicated severe anemia, with a RBC count of 2.48 × 10^12^/L and HGB of 48 g/L. An abdominal CT scan showed multiple round, cystic, hypodense shadows of varying sizes in the retroperitoneum and mesentery, with the largest measuring 2.5 cm × 2.1 cm and a density of 10.3 HU, located in the left lower quadrant and a small amount of abdominopelvic effusion was also present (Figure 1D). A chest CT scan revealed increased lung field transparency, cystic translucent shadows scattered in both lungs, and bilateral pleural effusion (Figure 1E). To address the pleural effusion, closed drainage was performed on both sides of the patients’ chest, draining mild bloody chylous fluid. Cytopathological analysis confirmed that the fluid did not contain any LAM cells or other malignant tumor cells. Due to the patients’ shortness of breath, he was unable to cooperate with pulmonary function test. A peripheral blood whole-exome gene sequencing was performed, which did not reveal any pathogenic or potentially pathogenic gene variants related to the patient’s clinical presentation. However, other potential pathogenic genes, including PEX7 and SLC26A4, HMGCS2, PRKCD and MCMC4 of unknown significance were identified (Table 1). After a multi-disciplinary discussion and patient briefing, a new treatment plan was initiated on 18 October 2023, with the patient taking 1 mg of Sirolimus orally daily. During both hospital stays, the patient did not receive any adjuvant treatments, such as chemotherapy or radiotherapy, apart from the aforementioned methods. Following the initiation of Sirolimus treatment, the patient’s symptoms, including black stool, chest tightness, and shortness of breath, improved, and he was discharged on 23 October 2023. Monthly hospital visits were scheduled for the first three months post-discharge to monitor treatment efficacy, followed by monthly follow-up calls. Throughout the Sirolimus treatment period, his condition remained stable, without recurrence or drug-related side effects, such as mucositis, canker sores, and diarrhea.

**TABLE 1:** Results of whole exome gene detection.

Gene	Population frequency	Pathogenicity classification	Related diseases
PEX7	0.0007	Pathogenicity	1. Peroxisome biosynthesis disorder type 9B 2. Proximal limb punctiform chondrodysplasia type 1
SLC26A4	0.005	Pathogenicity	1. Often cryptogenetic deafness type 4 with enlarged vestibular aqueduct 2. Pendred syndrome
HMGCS2	0	Unclear meaning	3-hydroxy-3-methylglutaryl CoA synthetase type 2 deficiency
PRKCD	0	Unclear meaning	Autoimmune lymphocyte proliferation syndrome type III
MCMC4	0.002	Unclear meaning	Immune deficiency type 54

## Discussion

Lymphangioleiomyomatosis (LAM) is a rare tumor caused by the proliferation of smooth muscle cells around lymph nodes and interstitial lymphatics (7). It is a slowly progressive, systemic disease associated with cystic lung destruction, abdominal tumors, and chylous fluid accumulations due to infiltration of neoplastic LAM cells. Women of childbearing age are more likely to be affected, and most cases manifest as pulmonary lesions (8). The prevalence of LAM is extremely low, with only 3.4–7.8 cases per 1 million females reported in the literature (9). While there have been documented instances of gastrointestinal LAM, cases involving male patients remain exceedingly rare. One case has been reported in the literature about a young man with diffuse abdominal LAM who experienced symptoms for six months, including vomiting, weight loss, intermittent abdominal pain, bloating, and constipation (6). Goh et al. (5) documented a case of a female with LAM affecting the ascending, transverse, and descending colon, primarily presenting with constipation. Song et al. (10) reported a case of a young man diagnosed with ascending colon LAM who manifested with intermittent right upper quadrant pain. Despite these similar cases, instances of LAM involving the alimentary canal and manifesting as gastrointestinal bleeding are seldom reported in the existing literature. This study provides a comprehensive account of the presentation and progression of this uncommon gastrointestinal LAM in males, which can help raise awareness among clinicians.

Most scholars believe that LAM is caused by gene mutation, specifically in the tuberous sclerosis complex (TSC) gene. There are two forms of LAM: Tuberous sclerosis LAM (TSC-LAM) and sporadic LAM (S-LAM) (11). Among the LAM patients, S-LAM accounts for about 85% of LAM cases, while TSC-LAM accounts for 15%. Both forms involve mutations in the TSC gene (12), including mutations of TSC1 and TSC2. The TSC gene is a tumor suppressor gene located on autosomes and is widely expressed in the body. When this gene is mutated in LAM patients, it loses its regulatory function of the TSC gene, leading to overactivation of the mammalian target of rapamycin (mTOR) (13), a key regulator of cell growth and proliferation (14). This results in the abnormal proliferation of smooth muscle cells, also known as LAM cells. In this case report, a whole exome gene test was performed, but no LAM-associated gene mutations were detected. Mutations in PEX7 and SLC26A4 were identified, but these are associated with different diseases. The significance of mutations in HMGCS2, PRKCD, and MCMC4 is still unclear. While the theory that TSC gene mutations cause LAM is primarily based on studies of female pulmonary LAM (PLAM), it does not rule out the potential role of other gene mutations in male LAM. In this patient, TSC gene mutation was not detected, allowing us to preliminarily exclude it as a cause. The mutated genes that were detected, their known clinical significance, and potential related diseases are listed in Table 1. Further research is needed to determine if these gene mutations are linked to the onset of this patient’s condition.

Diagnosing LAM presents challenges. Typically, patients with LAM do not exhibit specific symptoms in the early stages, and many of their symptoms resemble those found in other lung conditions, such as asthma, chronic obstructive pulmonary disease (COPD), and bronchitis. The two most frequent clinical presentations of LAM are recurrent pneumothoraces and dyspnea (15). Respiratory symptoms are the first clinical presentation in most patients diagnosed with LAM (15–17). Less commonly, LAM may present with a chylous effusion, an abdominal or pelvic mass, coughing up blood, abdominal bleeding due to a renal angiomyolipoma, or as an incidental finding of lung cysts and abdominal tumors (4, 9, 18, 19). In our case, the patient experienced recurrent abdominal pain, intermittent black stool, dizziness, and fatigue for more than a month. Abdominal CT examination did not reveal any significant mass within the abdominal cavity. After completing a series of ancillary tests, the cause of persistent gastrointestinal bleeding remained unclear. Eventually, the pathological examination of the surgical excision specimen indicated that LAM-like lesions were observed in the submucosal layer of the pylorus, mesentery of the side of the lesser curvature of the stomach and small intestine. The immunohistochemical results showed Ki67 (+5%), D2-40 (+), CD-34 (+), Desmin (+), SMA (+). It has been reported in the literature that Desmin (+), SMA (+), and D2-40 (+) are features of the immunohistochemistry of LAM. Desmin and SMA are myogenic markers for smooth muscle-like cells, while D2-40 is a marker of lymphatic endothelial cells (20–22). After correlating the abdominal CT findings, which revealed cystic lesions in the abdominal cavity, with the microscopic pathology and immunohistochemistry features, this case was ultimately diagnosed as abdominal LAM.

Lombard (23) proposed that LAM cells metastasize through the lymphatic tract. These LAM cells in the lymphatic fluid block the lymphatic duct outlet, resulting in increased lymphatic pressure (23). This can lead to the dilation of lymphatic vessels, as observed in the current patient. The patient’s pathology sections revealed enlarged lymphatic vessels, and abdominal CT scans showed signs of cystic lesions. In the lungs, clusters or nests of LAM cells infiltrate the walls of vessels, disrupting these walls and ultimately causing bleeding into the alveoli (24). Based on these findings, it can be speculate that the proliferation of LAM cells infiltrating the walls of gastrointestinal vessels could cause bleeding at the corresponding sites, which may account for the patient’s gastrointestinal bleeding symptoms. Moreover, the nodular lesions identified in the removed stomach and duodenal tissue are a manifestation of LAM cells proliferating in clusters.

Recent studies have shown that LAM cells, which are present in the blood and body fluids of S-LAM patients, have the ability to metastasize and spread to other areas of the body. This suggests that these cells are capable of moving away from their original location and implanting in new sites (25). The biological behavior of LAM is similar to that of low-grade neoplasms, and it has been found to metastasize (26). LAM cells within the abdominal cavity can travel through the lymphatic system and reach the lungs, where they can evenly distribute to both the left and right lobes. Once in the lungs, these cells invade and infiltrate the lung tissue, forming uniform lesions on both sides (23). In this case report, LAM cells metastasized from the abdominal cavity to the lungs, resulting in lung injury. This was expressed as a diffuse distribution of thin-walled cysts cavity and chylothorax in both lungs, leading to symptoms such as chest tightness and shortness of breath.

A portion of LAM located in the abdomen may initially not respond well due to the inability to identify the cause. However, a definitive diagnosis can often be made through a pathological biopsy following surgery (5, 7). When LAM occurs without a tumor mass, there are no established standardized treatment protocols. In cases where there is gastrointestinal bleeding or intestinal obstruction, surgical intervention may be necessary (6). In this study, surgical intervention was required when the patient’s gastrointestinal symptoms did not improve with conservative medical treatment. However, after the surgery, the patient continued to experience intermittent black stools and symptoms of chest tightness and shortness of breath. This suggests that the surgery did not fully resolve the gastrointestinal bleeding, and the condition may have even worsened, as indicated by the emergence of pulmonary symptoms. As a result, new treatment options needed to be explored.

A study has reported that a female patient diagnosed with pulmonary and retroperitoneal LAM experienced significant improvement after taking 1 mg/day of Sirolimus orally. Her pulmonary symptoms notably improved after six months of treatment, and the retroperitoneal lymph node lesions were completely resolved following three years of regular therapy (27). Consequently, the same treatment regimen of orally administering 1 mg/day of Sirolimus was used to manage disease progression in the current case.

Currently, mTOR inhibitors, such as Sirolimus and Everolimus, are the primary clinical treatment for LAM (28). Sirolimus is a highly targeted small molecule that binds to an immunophilin called FK506 binding protein, forming a complex. This complex then interacts with mTOR, blocking the activation of kinases downstream of the mTOR pathway and reducing mTOR expression. This decrease in mTOR expression leads to a decrease in abnormal proliferation of LAM cells, thereby reducing the damage they cause to tissues and organs (29).

The American Thoracic Society/Japanese Respiratory Society (ATS/JRS) guidelines state that Sirolimus can improve lung function and quality of life in patients, as well as reduce the accumulation of angiomyolipoma, lymphangioleiomyoma, and chylous fluid. Sirolimus is recommended for LAM patients who have abnormal or decreased lung function. In cases where LAM patients experience symptomatic chylous fluid accumulation, such as chylous pleural fluid and ascites, it is advised to use Sirolimus before resorting to invasive treatments. These invasive treatments may include methods such as intermittent percutaneous drainage or the use of indwelling drainage devices (30). While the guidelines do not specify a precise duration for Sirolimus use, but in clinical practice, a low dose of 1–2 mg/day is often recommended. This dosage aims to maintain serum trough levels within the range of 5–15 ng/mL (31). However, studies have shown that using Sirolimus to treat LAM can lead to potential side effects, including mucositis, canker sores, diarrhea, nausea, hypercholesterolemia, acne-like rash, and lower limb swelling (32). To reduce the occurrence of drug-related adverse events, low-dose administration is recommended and may enhance the safety of long-term treatment with Sirolimus. It is important to note that discontinuing Sirolimus during treatment can result in further deterioration of lung function (30).

Currently, the clinical treatment effect of LAM is limited, and most treatment modalities can only delay the progression of the disease but cannot achieve a cure (28). During our follow-up period, we observed that the patient’s black stool episodes were still intermittent, but the frequency had significantly decreased compared to his initial hospitalization. Additionally, the patient reported a significant improvement in his chest tightness and shortness of breath symptoms, and his lung condition did not worsen. The patient is currently undergoing ongoing follow-up, and his condition remains stable with no recurrence observed. The effectiveness of the treatment will continue to be monitored throughout the follow-up process.

## Conclusion

This case report describes the progression and treatment of a male patient with LAM, which affects both the gastrointestinal tract and lungs. LAM is a rare disease characterized by progressive involvement of multiple organs and is often irreversible once it occurs. Early diagnosis of LAM is challenging, and symptoms like gastrointestinal bleeding can be easily misdiagnosed as an ulcer. However, if a pulmonary or abdominal CT scan shows multiple cystic lesions, the diagnosis of LAM should be strongly considered as a potential diagnosis, regardless of whether the patient is experiencing symptoms such as hemoptysis or gastrointestinal bleeding. A pathological biopsy should be performed as soon as possible to confirm the diagnosis. In addition, once LAM is confirmed, the first-line medication, Sirolimus, should be initiated as early as possible after excluding any contraindications. A combined approach of surgery and Sirolimus may be a more effective strategy for slowing the progression of the disease. This case analysis provides valuable insights for clinicians in diagnosing and treating gastrointestinal LAM. Clinicians should be particularly attentive to LAM in male patients, as pulmonary symptoms may not always be the initial manifestation. However, further research is necessary to gain a better understanding of the mechanisms of gastrointestinal LAM and to develop more effective treatment options.

## Data Availability

The original contributions presented in this study are included in this article/supplementary material, further inquiries can be directed to the corresponding author.
